# Ethical Considerations in Research with Genomic Data

**DOI:** 10.1080/20502877.2022.2060590

**Published:** 2022-04-28

**Authors:** Rachel Horton, Anneke Lucassen

**Affiliations:** Clinical Ethics, Law and Society group, University of Oxford

## Abstract

Our ability to generate genomic data is currently well ahead of our ability to understand what they mean, raising challenges about how best to engage with them. This article considers ethical aspects of work with such data, focussing on research contexts that are intertwined with clinical care. We discuss the identifying nature of genomic data, the medical information intrinsic within them, and their linking of people within a biological family. We go on to consider what this means for consent, the importance of thoughtful sharing of genomic data, the challenge of constructing meaningful findings, and the legacy of unequal representation in genomic datasets. We argue that the ongoing success of genomic data research relies on public trust in the enterprise: to justify this trust, we need to ensure robust stewarding, and wide engagement about the ethical issues inherent in such practices.

## The nature of genomic data

DNA has phenomenal storage potential by virtue of its compactness, stability, and quaternary code. Sequencing DNA to create computer files amenable to analysis therefore generates enormous volumes of data – one sequenced human genome takes around 200 gigabytes of storage (100 Genomes Project, 2018). Intrinsic in genomic data is the potential to identify the provider of the genome, and to make inferences (albeit often uncertain) about their traits or health. Each person has around four million points at which their genome differs from the reference human genome sequence (Auton *et al*. 2015). Many of these variations will have no known impact on health, but could be used to draw conclusions as to where some of that person’s ancestors may have lived. Some variations will be associated with traits or disease that might be affecting that person in the present, or that might manifest in the future for them, or their biological relatives. The medical significance of many genomic variations will be unknown or uncertain, and knowledge might advance in an unpredictable way. For example, genomic variants influencing susceptibility to COVID-19 have been present in people’s DNA all their lives but were given medical meaning by the emergence of SARS-CoV-2 (COVID-19 Host Genetics Initiative, 2021).

Genomic data are being collected in the context of a diverse data explosion, and in working with genomic data there is likely much to learn from how other data have been used (or misused). Genomic data have distinct qualities in their ability to link people with biological relatives; however, other data (such as Facebook data or phone location data) have comparable ability to situate people within a social family and network. Genomic data have some potential to predict disease, but so do other forms of data (for example, a study of Google search histories of patients presenting to an emergency department found that health-related searches doubled in the week prior to the visit (Asch *et al*. 2019). However, genomic data are unusual in the extent to which they are stable across a person’s life course; genomic data collected today will have enduring relevance to the provider and their relatives many years down the line, while other data that the same person might provide today (for example, from their purchases or internet searches) will more rapidly dwindle into being of purely historical interest.

The variety of inferences that can be made from genomic data are also unusual, as so many different questions may be asked of the same data, from recent ancestry to likely traits, to disease risks over a lifetime. The Facebook-Cambridge Analytica scandal highlights the need to be mindful that the scope of consent for collection of the original data may not be sufficient when considering new research questions. The harvesting of data from 87 million Facebook users in order to target political adverts resulted in outcry partly because most of these users had not in any sense been asked for their consent, but also because even for those few who had (from a tick-box perspective agreed to data collection), their data was being used for purposes very different to what they expected (Nature, 2018).

### Genomic data in medical research

Genomic data are a hugely important resource in medical research. However, in order to give genomic datasets value, other data must be tied to them, because in order to learn how genomic variation impacts on health, researchers need both genomic data (genotype), and information as to what health issues – if any – the providers of those data have encountered (phenotype). Detailed, accurate phenotyping and durable and regularly updated links between genotypic and phenotypic data increase the usefulness, but also the sensitivity, of datasets.

Various forms of medical research involve genomic data, with rare disease diagnosis perhaps representing the greatest success story so far. In this context, genomic data are typically analysed with the aim of finding a single underlying genetic cause for a health condition which is typically severe, and often early-onset. For example, the Deciphering Developmental Disorders (DDD) study analysed genomic data from children with previously undiagnosed rare conditions and their parents and achieved a diagnostic yield of 40% (Wright *et al*., 2018). The Solve-RD consortium is connecting rare disease experts across Europe to share and jointly analyse genomic data for people with rare conditions with an unknown, but likely, genetic cause (Zurek *et al*., 2021). The success of genomics in investigating rare conditions is likely partly because many of those now diagnosed are caused by a single, highly disruptive variant that is often *de novo*, and comparison with parental genomic data is a very effective way to bring such variants to the fore. Rare disease researchers are increasingly casting the net wider, for example, looking outside the coding region of genes (Whiffin *et al*., 2020), but remain at an advantage as they know that (for the most part) there is a needle to find in the haystack, whereas common disease can rarely be attributed to a single variant.

Genomic data are also used in research into common diseases. Sometimes, researchers take an approach similar to that used for rare disease, by analysing genomic data from families with very severe or early-onset forms of the disease with the aim of finding strong genetic risk factors that might represent therapeutic targets. However, often the intention is to identify a slew of genomic variations which each make a tiny contribution to an individual’s risk of developing a particular disease but in combination say something more significant about risk. These might then be candidates for inclusion in polygenic risk scores aiming to identify people at high or low risk for common diseases, though some challenge whether such scores will ever be sufficiently discriminatory to usefully augment existing successful screening programmes (Wald and Old, 2019, Sud *et al*., 2021).

### Key ethical issues regarding genomic data

#### Limitations of consent in the context of genomic data

As discussed earlier, genomic data are inherently identifying and have (limited) potential to predict current or future health issues for both the provider of the genome, and their biological relatives. Common political discourses around genomic data may mean that people may have high expectations as to what might be gained from research into their data, whether for them personally, or for society as a whole (Ballard *et al*., 2019). This creates complexities around the consent process for the generation, storage, and future use of such data (Horton and Lucassen, 2019a).

Firstly, in providing genomic data for research, a person inevitably also provides genomic data from their biological relatives, regardless of what these relatives themselves might want. To give an extreme example, a person with an identical twin could in essence provide their twin’s entire genetic code, even if the twin was vehemently opposed to participating in research. More commonly, people will be contributing 50% of their parent, sibling, or child’s genetic code to projects amassing genomic data, more likely without their knowledge than explicitly against their wishes. The inextricability of a person’s genetic code from that of their biological relatives should not prevent research using genomic data, but does emphasize the importance of having robust and trustworthy mechanisms to ensure that data are used and protected in a thoughtful and ethically defensible way. This is particularly important given that a recent survey of 36,268 people across 22 countries exploring public attitudes to genomic data sharing found that willingness to donate one’s DNA and health data for research is relatively low (Middleton *et al*., 2020). It also highlights the importance of encouraging people to talk to their relatives when considering contributing their genomic data to research (or indeed to any repository, for example, those of direct-to-consumer genetic testing companies).

Secondly, current political discourse around genomic data in research tends to be strongly optimistic, and may create inflated expectations as to what genomic research in the near future is actually likely to deliver. For example, the UK policy paper *Genome UK: the future of healthcare* sets out ‘*an ambitious and compelling vision for how we will create the most advanced genomic healthcare ecosystem in the world*’ and discusses how we must use genomics to ‘*re-focus the healthcare system more towards prevention, earlier detection of disease, and promotion of wellbeing, rather than simply the diagnosis and treatment of illness*’ (Department of Health and Social Care, 2020). As discussed earlier, the role of genomic data in preventing or predicting common disease is largely unproven and has at times been significantly overstated (Wald and Old, 2019, Sud *et al*., 2021). Analysis of genomic data has proven utility in diagnosing rare disease, but many people with rare genetic conditions remain undiagnosed (Wright *et al*., 2018). The climate of expectation around the insights that can currently be gained from genomic data may undermine people’s ability to weigh the benefits and risks of participation in projects involving analysis of such data (Klima *et al*., 2014, Ballard *et al*., 2019).

Thirdly, future uses and potentials of genomic data are uncertain, but these data will have enduring relevance to the providers and their families, and unpredictable monetary value for the data holders. The consequences of contributing genomic data are difficult to forecast, meaning that consent from data providers cannot be other than broad and open-ended. Ideas such as dynamic consent, where people can revisit and review consent decisions over time, go some way to mitigate this (Teare *et al*., 2021) but still fall short – the potential outcomes from and uses of genomic data are so myriad that questions it might have been helpful to ask earlier are sometimes only clear in retrospect, and cannot be asked without revealing something of the situation that has now arisen (Horton *et al*., 2019). The limits of consent place a huge responsibility on people holding genomic data to justify any action they might take with these data – rather than acting as a *carte blanche*, the necessity of asking consent in broad terms increases the need to consider very carefully how to work with genomic data in a way that respects the reasons for which it was contributed. Another challenge is how to draw uncertainties as to the future of genomic data into consent conversations, in a way that seems meaningful to prospective providers.

#### Genomic data sharing and stewardship

Research drawing on genomic data has already led to improvements in medical care for many people. For example, many previously unrecognized genetic conditions have now been identified and described, so many more people now have a genetic explanation for previously unexplained health problems. Reaching a genetic diagnosis brings many benefits – the potential opportunity to predict and manage associated health issues that might arise; new reproductive options; the chance to link with other families affected by the same genetic condition; an end to numerous potentially invasive investigations aiming to establish why medical problems have happened (Wright *et al*., 2018). Research with genomic data has also led to improvements in cancer care, for example identifying potential treatment targets, and giving new options for monitoring treatment response (Berger and Mardis, 2018).

Data sharing is essential for achieving these benefits: genomic data are given clinical meaning by (large scale) comparison (Wright *et al*., 2019, Johnson *et al*., 2020). For example, databases of natural genetic variation (e.g. gnomAD), databases of variation thought to contribute to disease (e.g. HGMD), and data from relatives might all be brought to bear in interpreting a patient’s genomic data. As genomic databases enlarge and improve, so too does the ability to make diagnoses. For example, in 2014 the DDD project reported finding a diagnosis for 27% of the first 1,133 families recruited; reanalysing the same data in 2017 took the diagnostic yield to 40%. Most of the additional diagnoses were due to novel developmental disorder-associated genes discovered in the intervening three years (Wright *et al*., 2018).

Evidently, pooling of genomic data has great benefits – Johnson *et al*. make a convincing case that a genomic dataset held by the NHS could be considered a public good (Johnson *et al*., 2020). They draw on considerations of fairness to argue that at least those patients who benefit from genome sequencing have an ethical obligation to share their health information. This highlighting of the potential good that can arise from genomic data sharing is very welcome; it is also important to emphasize that harvesting data then not making good use of them is not morally neutral. We need to move to a position where thoughtful sharing of genomic data is expected, encouraged and facilitated.

This presents a challenge for genomic data holders as from an ethical perspective there is no simple ‘default’ position regarding genomic data sharing: they need to actively make choices about what data they should share, and what data they should withhold. In making such choices, they need to weigh the usefulness of making the data available against the risks of harm from doing so. The decision to share or not to share is a graded one – the quantity of genomic data shared per provider, the phenotypic data they are coupled with, and the audience with whom they are shared all need consideration.

Wright *et al*. make the case for a principle of proportionality in genomic data sharing – the depth of the data (what is shared) should be weighed against the breadth of sharing (with whom it is shared) (Wright *et al*., 2016). This curation is important as removing obvious identifiers from genomic data is not sufficient to protect providers against re-identification. For example, cross-referencing a ‘de-identified’ genome sequence with a genealogy database might identify biological relatives of the provider, enabling inferences to be made as to their identity – this technique was used by police to identify the Golden State Killer (Guerrini *et al*., 2018) (while this case can be seen as of overall benefit to society, there will be other examples where the balance is less clear). Re-identification is made more difficult by limiting the quantity of data shared per person, for example sharing a small number of variants per individual, rather than swathes of sequence. However, providing sufficient detail and context to ensure that data have the potential to be clinically useful to others, while avoiding providing so much detail that re-identification becomes an appreciable risk, requires expertise and investment.

What would facilitate trustworthy stewardship of genomic data? It is tempting to expect regulation to determine who should have access to which genomic data and when. However, while this may give the illusion of security, unless thoughtfully done it either disclaims responsibility for decisions to share by branding them as something that the provider ‘consented to’, or risks stifling worthwhile research by encouraging automatic refusal to data sharing, leaving little room for nuanced decision-making. A particular difficulty with stewarding genomic data is that decisions relating to their use often need to be taken within the constraints of current governance systems that treat ‘research’ and ‘clinical care’ as separable and distinct activities (Horton and Lucassen, 2019b). However, many activities that draw on genomic data sit in a hybrid space between the two. For example, many research projects using genomic data from patients with rare genetic conditions have a primary aim of finding clinical diagnoses for these patients (Wright *et al*., 2018, Zurek *et al*., 2021). Until the hybrid space between research and clinical care is recognized and valued, holders of genomic data risk being placed in situations where regulation and governance obscure rather than incentivise ethical practice. As Onora O’Neill points out, ‘*incompatible or barely compatible requirements invite compromises and evasions; they undermine both professional judgement and institutional autonomy*’ (O’Neill, 2002).

#### Data generation and entrenching inequality

Databases of natural genomic variation are vitally important to the practice of good genomic medicine. Genomic data from people with health problems are filtered against such databases, on the premise that genomic variations present in multiple ‘healthy’ people are unlikely to be responsible for severe disease. Historically, most people whose genomic data are represented in databases of natural variation have been of European ancestry. This means that the filtering process essential to shortlisting variants that might influence disease works more efficiently when the genome under investigation comes from a person of European ancestry. Similarly, most participants in genome-wide association studies (which look for links between genetic variants and disease) are of European ancestry; any associations then detected will be most applicable to people of European ancestry.

Historical under-representation in genomic databases directly contributes to disparity in clinical care. For example, Manrai *et al*. found that five genomic variants previously thought to cause hypertrophic cardiomyopathy were unexpectedly common in publicly accessible genomic databases. They hypothesized that these variants had been wrongly classified as being linked to cardiomyopathy, because databases of natural genetic variation that had been used as ‘controls’ in interpreting the significance of these variants had not included people from a sufficient range of ancestral backgrounds. The supposed extreme rarity of the variants had been taken as evidence that they were likely to be disease-causing; in fact, the variants were quite common in particular ancestral groups (with a minor allele frequency >1%). The authors reviewed patient records from a large genetics laboratory and found that multiple patients had received reports saying that these variants were disease-causing: all of these patients were of African or unspecified ancestry (Manrai *et al*., 2016).

Unequal representation of genomic variation across ancestral groups therefore increases the risk of misdiagnosis for people from underrepresented ancestral backgrounds, but also reduces the chance of achieving a correct diagnosis. Petrovski and Goldstein analysed genomic data from 5,965 people participating in various studies at the Institute for Genomic Medicine, using publicly available genomic databases to exclude common variants, leaving a shortlist of variants of potential interest. They found that for people of European ancestry, this shortlisting process was more effective than for people with non-European ancestry (Petrovski and Goldstein, 2016), meaning that reaching a diagnosis would likely be easier as there were fewer variants needing in-depth review. Caswell-Jin *et al*. illustrated the issues created by looking at results from gene panels assessing hereditary cancer risk: people who reported their race as white and their ethnicity as neither Hispanic nor Ashkenazi Jewish had a lower rate of variants of uncertain significance in their genetic test reports (Caswell-Jin *et al*., 2018).

These issues have been evident for many years, and efforts are being made to redress them. However, the legacy of underrepresentation in genomic databases has an ongoing impact on clinical care. Drives to make population genomic databases more inclusive and representative are clearly very important, but there are ethical questions to be considered in this process. For example, encouraging people from underrepresented ancestries to participate in genomics databases is important to ensure a more equitable future for genomic medicine, but there is also a need to acknowledge past abuses and the impact that these may have on willingness to participate (Popejoy *et al*., 2018). Another consideration is that deepening the diversity of genomic databases will likely increase their monetary, as well as their clinical value – what risks does this create around exploitation, and how should these be managed?

#### Constructing ‘results’ from genomic data

Within each person’s genome will be variations that might impact on their health, either now or in the future. The clinical significance of many of these variations will be minor or uncertain, but some people will have genetic variations which if known about, would make a substantial difference to their medical care. In generating genomic data, these variations become potentially visible. However, as mentioned above, each person will have around four million variations catalogued in their genomic data (Auton *et al*., 2015) – the filters which scientists apply in analysing these data will determine which variations float to the surface for further scrutiny. What responsibilities might this create?

Thorogood *et al*. reviewed laws and policies applying to communication of ‘results’ from research with genomic data from 20 countries and found that rules were often contradictory and inconsistently interpreted (Thorogood *et al*., 2019). The variety and complexity of regulation in this area illustrate how challenging it is to work out an ethically sound approach to constructing results from genomic data. For example, how should the context of the research project make a difference to what should be searched for and valued as a result? Use of genomic data for genome-wide association studies (which look *en masse* at genomic data from many patients) likely creates different responsibilities to analysing genomic data from specific families with undiagnosed conditions, but how might these responsibilities be enacted in practice?

Interrogating genomic data ‘because it is there’ has resource implications. Dorschner *et al*. (2013) screened 1,000 people from the NHLBI Exome Project for pathogenic variants in 114 genes selected by an expert panel because they were associated with adult-onset conditions where screening and/or treatment might be available. They identified 239 variants classified by the Human Gene Mutation Database as disease-causing that then went for expert review – on average, this took 23 min per variant to check how common the variant was and review the literature relating to it. Some variants required further discussion to reach a consensus regarding their pathogenicity (Dorschner *et al*., 2013). Ultimately, only 7.5% of the 239 variants were decided to be pathogenic or likely pathogenic.

Searching for and reporting health-related findings from genomic data is not simply a matter of running the data through a quick programme that spits out ‘results’ – it takes time and expertise to ensure that the findings are clinically sound, and this takes resources that then cannot be used for other things, for example forwarding the primary aim of the project collecting the data. Arguably, in the context of a publicly funded health service, searching for extra health information in genomic data might also exacerbate inequality by essentially offering a barrage of opportunistic genetic tests to people with a low chance of having the genetic conditions in question. This might lead to expensive long-term screening or preventative treatments being initiated, reducing the resources available to people with a much greater *a priori* risk of disease. In developing NHS screening programmes, costs and benefits are rigorously scrutinized, and it is interesting to note that none of these programmes yet involve searching for variants in the genes where the American College of Medical Genetics and Genomics recommends opportunistic analysis for people having genomic tests (Miller *et al*., 2021).

At the level of an individual participant, finding out such health information from their genomic data might be useful, but this does not necessarily justify diverting resources to seek it out. However, this does create potential responsibilities if scientists and clinicians become aware of genetic factors that likely represent a high risk of serious but preventable disease in an individual’s genomic data. Currently, many holders of genomic data have research pipelines that actively filter genomic variants in a way that minimizes ‘incidental findings’, yet they simultaneously apply ‘additional finding’ gene panels to the same data that actively search for and pluck out health-relevant information not related to the condition under study (Caulfield *et al*., 2015). Such plans illustrate how those working with genomic data on the one hand often try not to ‘see’ unexpected health-relevant information, yet concomitantly imply value in seeking such information. ‘Additional findings’ panels have the benefit of allowing some control over what sort of health information is looked for, but risk creating the illusion of similar control over what is not looked for, and resultant discomfort when something is found that was not ‘supposed’ to be found. Arguably, it would be more consistent (and a better use of resources) to engage with the few cases where health-relevant genomic information is incidentally detected during research, than to create situations where such information is both suppressed and sought within the same project.

#### Environmental impact of genomic data research

A further ethical consideration in genomic data research is the environmental impact of the storage and use of those data. DNA itself is an exceptionally efficient storage material: in 2014, Goldman *et al*. reported storing computer files as DNA with a storage density of 2.2 petabytes per gram (Goldman *et al*., 2013) (one petabyte can store enough MP3 files to play new music continuously for 2000 years (BBC Bitesize, 2021)). However, storing, maintaining and processing computer files which record DNA sequence, consumes a lot of energy (Lucivero, 2020).

Samuel and Lucivero highlight the need to move towards an ethics of environmental sustainability in open science, and identify key challenges which are relevant to genomic data research (Samuel and Lucivero, 2020). Firstly, the environmental impact of genomic data research is hard to quantify, and secondly, this impact must be weighed against the social value of such research. They also make the point that unless the energy cost of genomic data research is widely acknowledged as needing mitigation, there is limited ability for any one actor to reduce the environmental footprint of their work with genomic data. Environmentally appropriate choices around data processing and storage may cost more than less sustainable options, so individual researchers may be comparatively disadvantaged by making environmentally aware choices if these are not backed by funders and research institutions.

### Creating a future for research using genomic data

Above, we have outlined that research with genomic data can create various ethical issues. However, such research has important potential to advance knowledge and improve medical care. What is needed to create an environment where research with genomic data can flourish in an ethically defensible way? We argue that firstly, it is important to have wider societal discussions regarding these issues – a shared understanding of the complex, uncertain and enduring nature of genomic data would both bolster the integrity of consent, and make space for discussions as to what should happen where consent cannot give answers. Secondly, we need trustworthy and coherent systems governing work with genomic data.

Middleton *et al*. are exploring attitudes towards genomic data sharing in the *Your DNA Your Say* project, a survey encompassing over 36,000 people across 22 countries. They report that 64% of people regarded themselves as ‘unfamiliar’ with genetics, and the majority of participants were either unwilling or unsure about donating their anonymous DNA and medical information for use by researchers (Middleton *et al*., 2020). Ongoing genomic research is contingent on data sharing being considered acceptable so clearly this finding is concerning. The authors highlighted that trust was consistently associated with willingness to donate data; Milne *et al*. explored this further and found that providing transparent information about who will benefit from data access was endorsed as the most important measure to engender trust (Milne *et al*., 2021).

This work highlights two key areas where potential solutions risk undermining each other: low familiarity with genetics and genomics among general publics; and the need to provide accurate information as a key requirement for trust in initiatives using genomic data. The *Your DNA Your Say* team discuss the effects of this tension in designing their survey (Middleton *et al*., 2020) – a question asked about donating ‘anonymous DNA’, partnered with a glossary explaining that ‘*It is questionable as to whether DNA information can ever be truly anonymous as our DNA code is unique to us and thus, in itself, could be used to identify us. However, in the circumstances we are exploring here, by making DNA and medical information ‘anonymous,’ we mean detaching personal identifiers from it*’. They explained that they had initially used the term ‘de-identified’ when designing the survey, but from pilot work, it became clear that people did not readily understand what this meant. This illustrates a quandary in engaging people over data use – accessible language is important for people to engage with issues in the first place (anonymous vs de-identified), but then clarification is given as what actually happens (de-identified does not perfectly equate to anonymous) – how can we achieve this without undermining trust (it said my DNA was anonymous when it wasn’t)?

Clearly, trust is a pre-requisite for future research with genomic data – without trust, people will be unwilling to provide the DNA samples and phenotypic data on which the enterprise relies. Currently, research projects are often able to capitalize on the trust that many people have in research and clinical institutions. For example, various participants in the UK 100,000 Genomes Project described how they did not recall some of the decisions they had taken at the time of joining and had not understood certain aspects of the project, but were comfortable with this because they trusted that health professionals and the project team would act in their interests (Ballard *et al*., 2020). In order to maintain this trust, we argue that it is important to be honest as to how trustworthy aspects of research with genomic data can actually be. For example, rather than saying ‘your data will be kept anonymous’ and caveating this separately, maybe we should say ‘your data will be kept as anonymous as we can make them’. Rather than saying ‘your data stay in the data centre’ we should say ‘we work hard to ensure that your data stay in the data centre’. This might put some people off participating, but if that is because they become aware that our ability to protect data has limitations, and so are able to decide that they personally are unwilling to take that risk, surely that is a good thing?

Sheehan *et al*. report on a ‘public ethics’ coproduction activity, where eleven members of the public and two academic ethicists considered together what trust and trustworthiness are in the context of sharing patient data for research within the NHS. They note that ‘*we are often told that it is a matter of trust: we need to trust, we need to build trust, we need to restore trust*’, but make the point that more sustained reflection is needed as to what we mean by ‘trust’ and ‘trustworthiness’, and this should affect policy around data sharing. They highlight the need to distinguish between reliability and trust, writing that ‘*Particularly in cases in which consistent and guaranteed performance is required, we may be better off relying rather than trusting. For example, the data systems and infrastructure which house patient data should be as secure as possible as well as being as robust and well curated as possible. We want assurances that these systems function in a way that protects patient data from misuse, error and corruption. Having such assurances is not a matter of trust or trustworthiness, but one of reliability*’ (Sheehan *et al*., 2020).

The authors also reflected that calls for accountability, openness and transparency are a common response to worries about trust, but that this suggestion ignores Onora O’Neill’s argument that in order for someone to trust, there has to be something ‘to’ trust. Ever more detailed accounts of processes may actually erode trust by removing people’s opportunities to trust (O’Neill, 2002). Accountability, openness and transparency may demonstrate reliability, and by doing so provide grounds for people to trust, but trustworthiness goes beyond reliability alone – it also involves having appropriate values and commitments (Sheehan *et al*., 2020).

For a future where research with genomic data is facilitated and ethically robust, we need public support (or at least neutrality) regarding such work, but we also need governance systems that appreciate the issues inherent in working with these data. People researching genomic data are not making decisions in a vacuum and Research Ethics Committees play a key role aiming to ensure ethical oversight of projects drawing on genomic data. However, whilst this paper has focussed mainly on genomic data in the context of research, the separation from their use in other areas such as clinical or commercial practice is artificial – many activities relating to genomic data incorporate elements of all of these. This should create opportunity, but in practice it often creates problems as people working with genomic data struggle to comply with the complex regulatory requirements of different domains.

Fletcher *et al*. undertook a Delphi study exploring the impacts that regulation can have on health research through an iterative survey asking experts’ perspectives. In conclusion, they advocated continuing to steer regulation away from strict rules-based approaches towards principles-based regimes, allowing researchers, regulators and publics to co-produce regulatory systems serving core principles (Fletcher *et al*., 2019). They discuss how principles-based approaches give regulation more potential to adapt to changing contexts and new technologies, and also incentivise ongoing conversations between researchers and regulators as work progresses.

A principles-based approach has many positives, but only in tandem with engaging with publics about the issues inherent in working with genomic data: any principles by which research is governed will need social as well as legal ratification in order to succeed. Reflecting on the *care.data* experience, Carter *et al*. argue that ‘*although the infrastructure was in place, the activities were perfectly lawful, and a case had been made for the possible benefits that might be generated, the experience of care.data starkly exposes an enduring truism about the limits of law: legal authority does not necessarily command social legitimacy*’ (Carter *et al*., 2015). The proposed GPDPR scheme (an attempt to streamline collection of patient data from GP practices in England) has been pushed back due to similar concerns (O’Dowd, 2021).

One aspect that might enhance social support for research with data is ‘no surprises’ as to how the data are used – this was recently added to the Caldicott principles recommended for application by the NHS when using confidential information (Caldicott, 2020). However, awareness of health data usage is low: a 2016 survey of 2,017 people found that only 18% reported having heard ‘a great deal or a fair amount’ about how academic researchers are using health data (Ipsos MORI, 2016). Research also indicates that people tend to become more accepting of sharing patient data during the course of deliberative studies (Ipsos MORI, 2016, NHS, 2017). If we aim for there to be no surprises for the 82% of people who do not feel informed about health data use in research, what research with genomic data would be possible? Working closely with public contributors to develop principles for working with genomic data will increase their legitimacy and justifiability, but will not avoid surprises for people who are currently not aware of or not interested in issues to do with data use.

The perception of ethical conduct is relevant to people’s willingness to participate in research, for example, a survey of 800 people in Australia asking their expectations around genetic biobanks found that ethical conduct was highlighted as a major priority – more so than maximizing new healthcare benefits (Critchley *et al*., 2017). However, ethical conduct and perception of ethical conduct are not one and the same, as highlighted by Samuel and Farsides who explored the role of ‘ethics review’ within the 100,000 Genomes Project. They found that Genomic England’s ethical framework provides a genuine space for ethical discussion, but cautioned as to the need to ‘*[remain] vigilant to ensure that its desire to ‘window dress’ ethical issues to gain public support does not overshadow the need to* be *ethical*’ (Samuel and Farsides, 2018).

## Conclusion

In summary, research using genomic data has potential to improve health, but raises various ethical issues. Genomic data are complex, identifying, and enduring, and these features create challenges both for the people contributing them, and for the people working with them. The consequences of contributing genomic data and the insights that might be gained from these data are difficult to forecast, meaning that people’s consent to use of their data in research cannot be other than open-ended. Comparison with other genomic data is also intrinsic to their interpretation, meaning that judicious data sharing may advance healthcare for others, and unequal representation in genomic datasets risks exacerbating inequality. Frequently, holders of genomic data cannot simply turn to ‘consent’ as their justification for what is done with those data, they must consider and balance other factors such as what would best advance scientific knowledge and medical practice, and what would best protect the interests of the people who have contributed their data for study. However, in order for research with genomic data to flourish, a greater public engagement with data issues will be vital. Genomic data research is currently possible because sufficient people entrust researchers with their data. To maintain this, we need to ensure that protections around work with genomic data are worthy of that trust, and better involve people in recognizing and responding to the ethical issues raised by genomic data research.
